# Supplementary material for: Physiological Responses of Paulownia fortunei to Leaf Herbivory by Epicauta ruficeps: Nitrogen Assimilation, Porphyrin Metabolism, and ROS-Driven Antioxidant and Phenylpropanoid Responses
Source: Plants (Basel). 2025 Nov 30;14(23):3659. doi: 10.3390/plants14233659 (PMC12694431; doi:10.3390/plants14233659)
Supplement: Supplementary file 1 [file plants-14-03659-s001.zip › plants-3979346-supplementary.pdf]

Table S1.Reagent kit information and calculation formulas

Kit Name	Art.No.	Manufacturer	Formula for calculating content/activity/production rate	Additional remark
H_2O_2	H2O2-2-Y	Comin Biotechnology (Suzhou, China)	Content=1.34×(ΔA-0.0006)÷W	ΔA=A445 Control tube （CT）-A445 Blank tube（BT）<br><br>W=0.1g
O2·-	SA-2-G	Comin Biotechnology (Suzhou, China)	Production rate=7.44× (ΔA +0.0027)÷W	ΔA=A530 Tset tube （TT）-A530（BT）<br><br>W=0.1g
SOD	YX-C-A500	Sinobestbio<br><br>Biotechnology<br><br>(Shanghai, China)	Percentage inhibition =（ΔA560（BT）-ΔA560（TT））÷ΔA560（BT）*100%<br><br>Activity=11.4*PI÷（1-PI）÷W	ΔA560（BT）=A560（BT-NO.1）-A560（BT-NO.2）<br><br>ΔA560（TT）=A560（TT）-A560（CT）
CAT	YX-C-A501	Sinobestbio<br><br>Biotechnology<br><br>(Shanghai, China)	Activity=678*ΔA240÷W	ΔA240=A240（0S）-A240(60S)<br><br>W=0.1g
POD	POD-2-Y	Comin Biotechnology (Suzhou, China)	Activity=2000×ΔA÷W	ΔA470=A470(60S)-A470(120S)
APX	APX-2-W	Sinobestbio<br><br>Biotechnology<br><br>(Shanghai, China)	Activity=1.79*(ΔA290（TT）-ΔA290（BT）	ΔA290（TT）=A290（10S）-A290（130S）<br><br>ΔA290（BT）=A290（10S）-A290（130S）
GPX			Activity=530*(ΔA340（TT）-ΔA340（BT）)	ΔA340（TT）=ΔA340(10S)-ΔA340(10S)<br><br>ΔA340（BT）=ΔA340(10S)-ΔA340(10S)

PAO	PAO-2-G	Comin Biotechnology (Suzhou, China)	Activity=166.67×ΔA÷W	ΔA=A550(0S)-A550 (30Min)
GS	GS-2-Y	Comin Biotechnology (Suzhou, China)	Activity=10.268×(ΔA-0.0008)÷W	△A=A （TT）-A （CT）
GOGAT	GOGAT-2-Y	Comin Biotechnology (Suzhou, China)	Activity=321×ΔA÷W	△A=A340（20S）-A340（320S）
GDH	GDH-2-Y	Comin Biotechnology (Suzhou, China)	Activity=643×ΔA÷W	△A=A340（20S）-A340（320S）
GSH		Sinobestbio Biotechnology (Shanghai, China)	Standard curve：y=0.0014X+0.1669 （R^2=0.9995） Content=y÷W	W=0.1g
AsA	YX-C-A300	Sinobestbio Biotechnology (Shanghai, China)	Y=0.246X+0.0845 （R^2=0.9995） Content=10y	X=A534 W=0.1g
Pro	PRO-2-Y	Comin Biotechnology (Suzhou, China)	Content=19.2×(A520+0.0021)÷W	W=0.1g
Glu	GLU-2-Y	Comin Biotechnology (Suzhou, China)	y = 0.0074x- 0.5255 Content=270.2×(△A+0.5255)÷W	Y=A570（TT）-A570（BT） X：Concentration of the sample solution W：0.1
Flavonoid	LHT-2-G	Comin Biotechnology (Suzhou, China)	Content=0.398×（ΔA -0.0007）÷W	ΔA=A510（TT）-A510 （BT） W=0.1g

Lignin	MZS-2-G	Comin Biotechnology (Suzhou, China)	$y=0.0694x+0.0068$（$R^2=0.9889$）<br><br>Content=0.0294×(ΔA-0.0068)÷W	ΔA=A280（TT）-A280 （BT）<br><br>W=0.1g
